# Fat-free mass and resting metabolic rate are determinants of energy intake: implications for a theory of appetite control

**DOI:** 10.1098/rstb.2022.0213

**Published:** 2023-09-11

**Authors:** Mark Hopkins, Catherine Gibbons, John Blundell

**Affiliations:** ^1^ School of Food Science and Nutrition, Faculty of Environment, University of Leeds, Leeds LS2 9JT, UK; ^2^ School of Psychology, Appetite and Energy Balance Research Group, Faculty of Medicine and Health, University of Leeds, Leeds LS2 9JT, UK

**Keywords:** fat-free mass, resting metabolic rate, energy intake, obesity

## Abstract

Any explanation of appetite control should contain a description of physiological processes that could contribute a drive to eat alongside those that inhibit eating. However, such an undertaking was largely neglected until 15 years ago when a series of independent research programmes investigated the physiological roles of body composition and appetite. These outcomes demonstrated that fat-free mass (FFM), but not fat mass, was positively associated with objectively measured meal size and energy intake (EI). These findings have been accompanied by demonstrations that resting metabolic rate (RMR) is also positively associated with EI, with the influence of FFM largely mediated by RMR. These findings re-introduce the role of drive into models of appetite control and indicate how this can be integrated with processes of inhibition. The determinants of EI fit into an evolutionary perspective in which the energy demands of high metabolic rate organs and skeletal tissue constitute a need state underlying a tonic drive to eat. This approach should lead to the development of integrated models of appetite that include components of body composition (FFM) and energy expenditure (RMR) as tonic biological signals of appetite alongside other traditional tonic (adipose tissue derived) and episodic signals (gastrointestinal tract derived).

This article is part of a discussion meeting issue ‘Causes of obesity: theories, conjectures and evidence (Part I)’.

…we must accept, the general principle, that people do not all eat, and do not all require, the same amount of food [1, p. 123]

## 1. Background

Until recently, neither the body's lean tissue (fat-free mass (FFM)) nor basal energy expenditure (resting metabolic rate (RMR)) had been considered to play major roles in the expression of human appetite. However, within the last decade convincing evidence has accumulated to demonstrate clear associations. Many researchers point to the work of Brobeck [2,3] and Kennedy [4,5] in the 1940s and 1950s as the starting point for investigations of the control of food intake (see [6] for an account of the early theories of appetite control). These classic studies on the hyperphagia produced by hypothalamic lesions established a line of thinking that linked the brain's control of food intake with body weight. Kennedy used the term ‘lipostasis’ to refer to the reciprocal relationship between food intake and body fat following ventromedial nucleus of the hypothalamus (VMN) lesions. A central feature of this idea was Kennedy's postulation of the existence of a blood borne signal that emanated from body fat and acted upon the VMN. Although Kennedy did not use the phrase ‘body fat regulation’, this idea was promoted by Mayer *et al.* [7] in a further classic paper, which made the case for the lipostatic hypothesis. This concept was embodied in the adipocentric approach to appetite control which posited that food intake was controlled in order to regulate adipose tissue, giving rise to the pursuit of the causes of body weight regulation or body fat regulation, which dominated the experimental study of appetite for half a century. This can be referred to as the *Zeitgeist* of this epoch of research.

During the same period in which Kennedy was conducting experiments on the food intake of rats, a group of physiologists and nutritionists were investigating the control of food intake in human subjects. These researchers were motivated by ‘*the desire to find out more about the mechanisms that relate intake to expenditure – what regulates appetite in fact*’ [8, p. 286]. This research involved the meticulous observation and measurement of 24 h dietary intakes and patterns of physical activity in (mainly) young men. The research was intended to reveal any relationships that existed between the energy expended in physical activities and the intake of food within any single day or over periods of several days [8,9]. These studies led the authors to conclude that: ‘*the differences in the intakes of food (of individuals) must originate in the expenditure of energy’* [8, p. 297]. Despite this line of thinking, which offered a plausible proposal for the study of appetite control, energy expenditure and its main determinants (such as FFM) have not featured heavily in models of human appetite until recently.

Implicit in such work are fundamental questions regarding the mechanism of appetite control and how these operate (if at all) within wider regulatory systems that influence energy balance and body weight. Classic homeostatic models of appetite have often positioned appetite simply as an outcome of energy or nutrient balance, but food intake is a volitional behaviour influenced by complex and individually subtle physiological, environmental, social and cultural factors [10]. Knowledge of these factors, and how their influence on eating behaviour changes under specific physiological (e.g. differing states of energy balance) or environmental situations (e.g. diets of varying energy density or macronutrient composition), is key in understanding their influence on body weight [11]. Indeed, one line of thought is that the homeostatic mechanisms of appetite control offer little protection against overconsumption in a modern environment rich in sensory and environmental cues for food intake [12]. An implication of this is that physiologic models of appetite control alone are unlikely to fully explain the causes of overconsumption and weight gain.

## 2. Body composition and the tonic drive to eat

A key feature of many theories of so-called body weight regulation is that the control of appetite is subservient to the maintenance of energy homeostasis and body weight, with negative feedback arising from the oxidation or storage of energy [13–15], fat [4,5], carbohydrate [16,17] or amino acid concentrations [18,19] suggested at some point as a signals for the drive or suppression of eating. Of these theories, homeostatic feedback concerning energy storage in adipose tissue via the action of leptin is now thought to play a role in the control of appetite and energy balance. Although evidence suggests that leptin plays a stronger role in the control of appetite during periods of energy deficit and weight loss than in resisting energy surfeit and weight gain [20,21], the role of leptin in communicating information concerning energy storage in adipose tissue has been interpreted as confirming a central role for body fat in overall appetite control. The prominence given to the regulation of body fat in the control of food intake is illustrated by statements in mainstream research such as ‘*There is compelling evidence that total body fat is regulated…when it is decreased reflexes restore it to normal…..when it is increased reflexes…elicit weight loss….’food intake is an effector or response mechanism that can be recruited or turned off in the regulation of body fat*’ [22, p. 5]. The situation changed when a number of research laboratories including the University of Leeds, the Rowett Research Institute in Aberdeen, the National Institute of Health/National Institute of Diabetes and Digestive and Kidney Diseases (NIH/NIDDK) in Phoenix and the University of Ottawa developed research approaches that included the concurrent measurement of body composition, resting energy expenditure, objective measures of food intake over 24 h often accompanied by measures of hunger and other appetitive states (e.g. [23]).

This approach permitted a direct test of whether food consumption was associated with fat mass (FM; as predicted by the adipocentric idea) or with FFM, body weight in general or body mass index (BMI). The approach was conceived as an investigation of the basis of a tonic drive to eat which was included in models of appetite control as a theoretical construct awaiting identification [24]. This work aimed to complement existing understanding of the tonic and episodic signals involved in the suppression of eating (e.g. post-prandial satiety) by stimulating renewed interest in the physiological processes that drive eating. A detailed account of the episodic nature of appetite control is beyond the scope of the present review, but readers are directed to previous reviews on these mechanisms [11,25–27] and their measurement [28,29]. Rather, the present review highlights recent research examining FFM and RMR as physiological sources of tonic appetitive signals which act alongside other tonic signals (e.g. the inhibitory influence of leptin) in providing slow modulation of appetite to reflect metabolic energy requirements (e.g. RMR) and longer term energy storage (e.g. adipose tissue) in day-to-day energy intake (EI). In doing so, we discuss the tonic appetite processes that link the physiological need for energy with the behaviour that satisfies these needs, rather than eating prompted by the hedonic aspects of food intake [12]. This work positions FFM and RMR as key features of tonic appetite control, but this should not be taken to imply that appetite and food intake are determined entirely by these factors. The overall expression of appetite reflects a balance between stimulatory and inhibitory processes, and by focusing on the physiological mechanisms that drive appetite in the present paper, we aim to complement existing understanding of the inhibitory processes of appetite [11,25–27].

Evidence presented in this review for this tonic drive stemming from FFM and RMR is primarily based on cross-sectional studies in weight stable individuals at or close to energy balance. This is important to note as studies suggest that the effects of FFM on appetite may depend on energy balance status, with a number of studies suggesting that FFM loss following energy deficit may act as an additional orexigenic signal that promotes increased EI or weight regain specifically following weight loss [30–34]. The reader is directed to a number of theoretical discussions on the potential effects of FFM loss on appetite [35–37]. If FFM loss does act as an orexigenic signal during weight loss, then strategies that attenuate its loss could theoretically help counter compensatory changes in appetite. While the composition of weight loss is modifiable [38], we are unaware of studies systematically manipulating FFM to examine whether attenuating FFM loss during weight loss alters appetite-related outcomes.

## 3. Body composition and energy intake—what is the evidence?

Over 10 years ago, Blundell *et al.* [39] specifically examined the relationships between body composition and objective measures of self-determined meal size and 24 h EI. It was demonstrated that EI was positively associated with lean mass, but there was no relationship with FM or BMI in a group of people with overweight/obesity closely monitored over a period of 12 weeks. This finding was confirmed in a study on a large group of people with obesity residing in a biological research facility and self-selecting all foods from an automated vending machine [40]. This research team used FFM index and FM index and showed that the FFM index was positively associated with EI, while a weaker negative association was seen between the FM index and EI. These findings have now been confirmed in studies from numerous independent laboratories under laboratory [23,41–43], residential in-patient [41] and free-living situations [44,45], and this effect has been replicated in new born babies [46], adolescents [47,48], older adults [49], individuals with overweight or obesity [39,50] and in several different ethnic groups [40,51] (see Blundell *et al.* [52] for a detailed review of supporting evidence).

Interestingly, the positive relationship between body composition and EI had formed the basis of an earlier study [43] examining the accuracy of dietary reporting in individuals with obesity. The authors reported that energy requirements were positively associated with lean mass (but not FM). A conclusion of the study was that ‘*The emphasis of research* *on obesity that focuses on the relationship between EI and fat is misplaced because EI appears to be a direct function of lean mass rather than adiposity’* [43, p. 324]. It is important to note that this study was published more than 30 years ago [43], but the importance of these findings to the control of appetite has been overlooked (presumably owing to the adipocentric nature of the Zeitgeist and the narrow focus on body fat regulation).

## 4. Body composition and hunger

Hunger can be defined as a subjectively expressed construct that reflects the motivation to eat [10]. It is a biologically useful and universally identifiable sensation prominently associated with behavioural acts of food consumption (see [10,53] for reviews). In this context, it is important to distinguish between hunger arising from the biological processes reflecting nutrient availability and energy needs (as discussed here) from eating promoted by a desire for the pleasurable taste of food e.g. homeostatic and hedonic appetite processes. Given the nature of hunger and its significance for human EI, it is theoretically important to examine whether or not hunger is associated with FFM.

**Figure 1.**
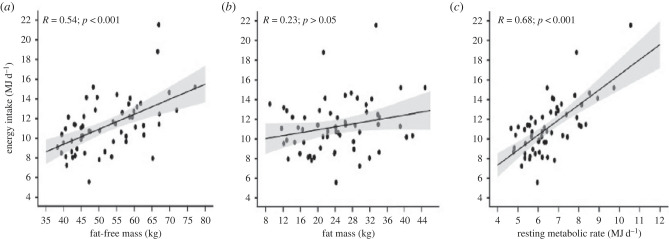
Associations between fat-free mass, fat mass and resting metabolic rate with energy intake. Adapted from Hopkins *et al*. [41].

The first observations were part of a study on day/night variation in normal weight participants in which a 10-point subjective rating scale was used to quantify subjective hunger at 30 min intervals across the day [54,55]. The pattern and strength of hunger was positively related to the body's FFM and inversely to FM in lean individuals [54], but no such associations were found in individuals with obesity [55]. Almost a quarter of a century later, the issue was investigated when the Visual Analogue Scale (VAS) was used to track daily hunger in a comprehensive 12 week study using a multi-level platform in participants overweight or with obesity [23]. A comparison of the top and bottom tertiles of FFM (balanced for sex) showed a significantly higher hunger throughout the day in those individuals with the greatest amount of FFM, a pattern also seen for tertiles of RMR [23]. Taken together these studies provide evidence for the proposition that FFM exerts tonic influence not only on quantitatively measured EI but also in the major subjective sensation associated with eating—hunger (figure 1).

Subsequently, Grannell *et al*. [50] investigated meal time hunger and food intake in people with severe obesity (BMI—44 kg m^−2^) and demonstrated that meal intake and pre-meal hunger was positively associated with FFM but not with FM. As noted by the authors these ‘*findings suggest that at* *the extremes of obesity FFM continues to promote hunger and EI*’ [50, p. 1], but the strength of this association was weaker in individuals with a higher BMI. These findings were supported by Casanova *et al*. [56] who reported that percentage body fat moderated the associations between FFM and free-living 24 h EI in 45 healthy weight and 48 individuals with obesity, with these associations becoming weaker at higher percentage body fat levels. When considered alongside similar findings [54,55,57], these data suggest that excess FM may disrupt the coupling between FFM and EI.

## 5. Fat-free mass *per se* or resting metabolic rate

Given that FFM is the main determinant of RMR [58], an important question arising from these studies is whether the reported associations between FFM and hunger or EI reflect the energetic costs of FFM or a specific molecular signal from FFM (or one or more of its constituent tissue organs). Cross-sectional studies have demonstrated that the effect of FFM on EI is mediated statistically by RMR [41,44] or total daily energy expenditure (TDEE) [57]. For example, Hopkins *et al*. [41] reported that the effect of FFM on EI was fully mediated by RMR, while Piaggi *et al*. [57] reported that TDEE accounted for 80% of the observed effect of FFM on EI. While these findings represent statistical rather than biological pathways, they suggest that FFM influences EI via its energetic contribution to RMR and TDEE. It should be noted however that skeletal muscle secretes numerous signals such as myokines that can purportedly influence EI directly via the hypothalamus or indirectly via effects on satiety hormone concentrations (see [59] for reviews). Although their role as candidate signals linking FFM to appetite has yet to be clearly established, it cannot be ruled out that specific molecular signals from skeletal muscle (or other organs) provide metabolic signals to the brain for the modulation of hunger and EI.

An important consideration is that FFM is a heterogeneous body compartment that comprises numerous individual tissue organs with wide ranging mass-specific metabolic rates which summate to whole-body RMR [60–62]. To date, there has been little attempt to integrate individual tissue organs and their mass-specific energy expenditures into homeostatic models of human appetite. While previous studies have typically used two-compartmental models of body composition to examine the associations between FFM and EI, Casanova *et al*. [63] analysed data from a study using whole-body magnetic resonance imaging in 21 lean healthy males to examine the association between FFM and EI at the tissue organ level. As would be expected, fasting hunger was associated with FFM (*r* = 0.39; *p* = 0.09), but interestingly the association between high-metabolic rate organ mass (e.g. the combined masses of the brain, liver, heart and kidneys; *r* = 0.58; *p* = 0.01) and fasting hunger was found to be stronger (although, not statistically different from one another). When the associations between individual tissue organs were examined, liver (*r* = 0.51; *p* = 0.02) and skeletal muscle mass (*r* = 0.57; *p* = 0.04) were found to be associated with fasting hunger. As these two tissue organs explained 17% and 21%, respectively, of the variance in RMR, it is plausible to suggest these associations with EI reflect their contribution to RMR. It should be noted that skeletal muscle was the largest and most variable component of FFM (by mass), and given the previously mentioned potential for skeletal muscle derived appetitive signals, future research should look to integrate measures of body composition at the tissue organ level alongside markers of their metabolic function with appetitive measures to examine the biological mechanisms linking FFM and its constituent tissue organs to appetite.

## 6. What is the mechanism?

The proposal that FFM and RMR act as tonic drivers of hunger and EI is of evolutionary significance since it provides a biological purpose for eating, which clearly favours survival. While adipose tissue acts as an important energy reserve, it is quite plausible to propose that the purpose of the drive to eat is not to regulate body fat but to provide energy to meet the energetic demands of vital organs and to maintain life and growth. In achieving this goal, body weight will obviously be preserved but that is a consequence and not the primary target.

The signals involved in sensing, integrating and translating the body's energy needs (arising from FFM) into eating behaviours are unknown. A role for lean or protein tissue in the control of appetite has previously been suggested, e.g. the aminostatic [18] and protein-static [19] theories of appetite, but outside periods of growth, evidence to support a role for amino-or-protein-static feedback in the control of food intake in humans is limited. Cerebral blood flow in the periaqueductal grey region has been shown to mediate statistically the association between FFM and hunger [64], but it can be questioned what type of mechanism could be identified that links FFM to the central brain regions involved on appetite, i.e. neural or endocrine (including cytokine or myokine) derived signals? Such a signal may originate from specific organs such as the liver or skeletal tissue or from an integrated feedback pathway from multiple tissue organs? Further, it can be considered at what level of physiological organization does this occur: cellular > tissue organ > whole-body metabolism? There has been rapid growth in our understanding of the signals involved in the crosstalk between peripheral tissue organs (including skeletal muscle) and the brain, with a number of cytokine and myokines thought capable of influencing EI via direct effects on the hypothalamus or indirectly via effects on satiety hormone concentrations [59,65]. However, their role as candidate signals linking FFM to appetite has yet to be clearly demonstrated in humans. Separate signalling pathways may also be involved in linking FFM to appetite and EI during conditions of energy balance and during energy deficit where tissue loss occurs, with studies reporting that FFM loss is associated with increased hunger during weight loss [31,66–68] and weight regain [33]. While the specific mechanisms again remain unknown, it has been suggested that muscle loss may influence EI via myostatin mediated changes in insulin-like growth factor 1 [59,65].

It is worth reflecting on the pathway linking FM with the brain which is now known to involve leptin and melanocortins. Moreover, following Kennedy's initial suggestion of a blood borne signal linking body fat with the hypothalamic VMN, it took over 40 years for leptin to be discovered and the pathway described. Identifying the link between FFM and EI with the brain will be a challenging task, and the mechanism is unlikely to be based on a single privileged molecule.

## 7. A model of appetite

**Figure 2.**
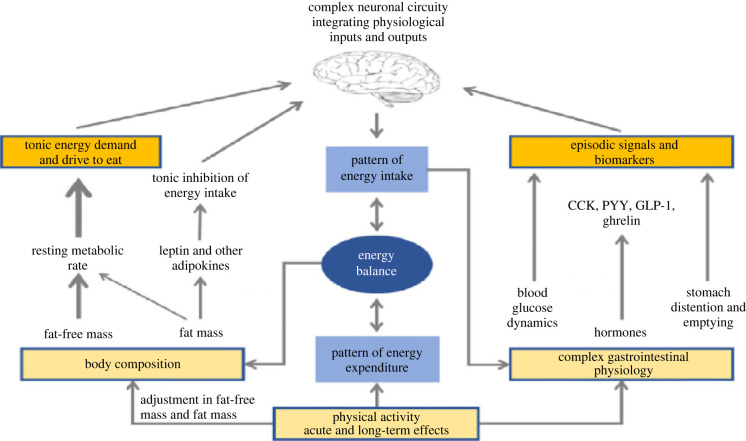
Theoretical model of appetite illustrating the tonic and episodic physiological processes of appetite control in which it is proposed that body composition influences appetite via both drive (fat-free mass and energy expenditure) and an inhibitory system (fat mass). Adapted from Blundell *et al*. [69].

For over 20 years, the Leeds approach to understanding appetite has been formulated around a division between tonic and episodic processes [24] as shown in figure 2. For clarity, the figure shows a structural separation of the two divisions, but in reality, the physiological processes interact, and this complexity is integrated by the brain. The episodic processes are concerned with the pattern of food intake and the events associated with antecedents of eating and with the complex cascade of post-prandial physiological sequelae induced by the act of food consumption (see [26,27] for reviews on these mechanisms). These events are quick acting, time limited and unpredictable (depending on behaviour). This aspect of appetite control reflects the omnivorous habit of humans and is hugely complicated given the diverse patterns of food consumption by people in different habitats across the planet, and by the thousands of different foods consumed. This aspect of appetite (right-hand side of figure 2) is distinct from the tonic processes which are the focus of this paper.

The left side of figure 2 shows the tonic processes reflected by body composition and RMR as described in §§3, 4 and 5 above. These processes are enduring and exert a more stable and uniform physiological pressure lacking the periodicity of the episodic processes. The model reflects the evidence for the dominance of the FFM and RMR in determining the drive to eat, with a smaller contribution from FM commensurate with its influence on RMR. We infer that this stimulatory effect on food intake is countered by the inhibitory influence of the leptin-melanocortin signalling pathway [70]. It can be envisaged that the complex signals generated through the tonic division are ‘interpreted’ by the brain and integrated with episodic signals in addition to being coordinated with relevant signals from ongoing metabolic processes. This model is not intended to be a detailed description of neurophysiology, but represents a framework for thinking about the integration of endogenous processes characterizing a person's physical makeup and metabolic activity with the complex and often chaotic activity of behavioural events encountered in a real world nutritional environment. It is hoped that this work leads to renewed interest in the processes that drive eating alongside those that suppress eating and, in particular, the aim is to disclose the underlying physiological mechanisms that translate energy needs into day-to-day food intake.

## 8. Interpreting the role of fat mass

While the focus of this review has been on the role of FFM in the control of appetite, it is important that this work should not be interpreted as suggesting that FM does not play a role in the control of appetite. Rather, acknowledging the role of FFM alongside adipose and gastrointestinal derived feedback is intended to provide a stronger account of the peripheral physiological signals involved in human appetite control. Although RMR may exert an effect on EI, the actual food consumed by an individual at any particular moment will be determined by a combination of FFM/RMR, FM and gastro-intestinal physiology, in addition to a wide range of sensory and cognitive variables, the nutritional composition of the foods and their accessibility, physical activity, time of day, environmental context and past history. Therefore, FFM/RMR alone cannot always predict the occurrence of an eating event nor what and how much is consumed. However, this explanation of the effect of energy demand can plausibly account for part of the individual variability between people in their drive to eat. The proposition that food intake is related to individual energy requirements (as also predicted by Widdowson [1] and Edholm *et al.* [8]) encourages us to place greater emphasis on the individual nature of food intake and eating behaviour, rather than on the average values.

In contrast with the consistent findings linking FFM to EI, studies that have directly examined the associations between FM and appetite or EI have shown these associations to be much more variable. For example, studies have shown that FM may have no significant relationship with EI [39,46,71], a negative relationship [41] or a positive relationship [40]. FM clearly has a more variable relationship with food intake than FFM does, and the nature of this relationship may differ depending on the level of adiposity. Negative associations between FM and EI have been observed in lean individuals [41,54], but studies in those with overweight/obesity often report no association between FM and EI [39,43,47,72].

**Figure 3.**
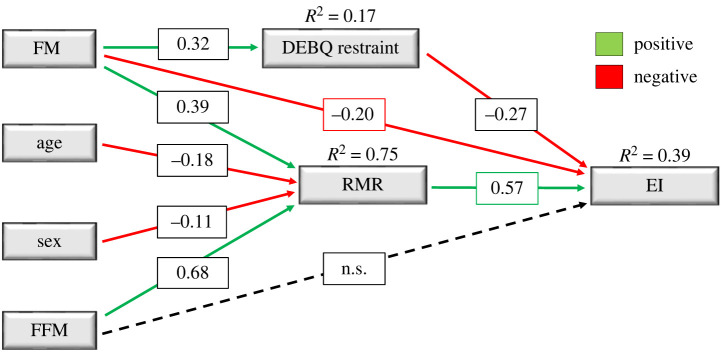
Path diagram of the direct and indirect effects of fat mass, fat-free mass, resting metabolic rate and cognitive restraint on mean daily energy intake. The effect of fat-free mass on energy intake was fully mediated by resting metabolic rate. The direct effect of fat mass was partially mediated by cognitive restraint and resting metabolic rate. FM, fat mass; FFM, fat-free mass; RMR, resting metabolic rate; DEBQ_R, restraint-Dutch Eating Behaviour Questionnaire and EI, energy intake. Adapted from Hopkins *et al*. [44].

These data imply that the effect of FM on EI will depend on the total amount of fat in the body. When the percentage of adipose tissue is small, FM appears to have a strong inhibitory effect—as shown in young lean males and females [41], and in the analysis comparing low and high amounts of FM in a group of adults [56]. A weaker negative association between FM and EI at higher body fatness is in line with the notion of leptin and insulin resistance [73,74], which may alter central and peripheral sensitivity to appetite-related feedback signals [75–77]. Our interpretation is that when the amount of FM is high, then leptin/insulin resistance increases and the inhibitory effect declines [56]. Hence people with obesity may not benefit from a strong inhibitory effect of FM on EI. Such findings raise the possibility that the relationship between FM and EI may be nonlinear [56], particularly in the extremes of obesity where factors such as insulin and leptin resistance might be greater. It is also important to recognize that psychological factors such as cognitive restraint are salient features of appetite that may also mediate the relationship between FM and EI. This can be seen in the path analysis shown in figure 3 [44], which is based on cross-sectional data in 242 individuals in which body composition (air displacement plethysmography), RMR (indirect calorimetry), eating behaviour (Dutch Eating Behaviour Questionnaire) and EI (6–7 day weighed-dietary records) were measured. In this path diagram, FM was shown to indirectly influence EI positively via its effect on RMR (albeit more weakly than FFM), but an indirect negative effect was also noted with EI which was mediated by cognitive restraint [44]. Therefore, considering the tonic appetite components in the model, the drive to eat will depend on the balance between the positive and inhibitory effects of FFM and FM. These effects will vary with the sex and age of the individual, the degree of obesity, the relative amounts of FFM and FM, and the state of energy balance (positive, negative or in balance).

## 9. Adipocentric hypothesis and the regulation of body fat

The findings presented above, and incorporated into the model of appetite, are clearly contrary to an adipocentric explanation and to the idea that food intake is controlled in order to regulate the amount of fat in the body. As postulated by Brobeck over 70 years ago the ‘*Regulation of fat storage appears to be a passive process, subject only indirectly to control. Presumably fatty deposits increase in size whenever energy intake exceeds total output, and decrease when the situation is reversed*’ [78, p. 317]. If this is true, then it follows, according to Brobeck, that the quantity of fat in the body is determined by mechanisms which regulate food intake, work (or activity) and temperature. Moreover, food intake is not solely controlled by body fat in order to regulate the amount of fat in the body. Even though fat storage may be a passive process, this does not mean that fat is inert with respect to EI. Indeed the landmark discovery of leptin [79] provided the key to the identification of a signalling pathway that linked FM to the brain. As noted by Brobeck [78] and earlier argued by Brody [80], for a given food intake body size is limited by physical laws. This argument renders unnecessary the need for a single controlled set point or upper and lower set points, since the body weight will reach a level determined by behavioural actions (EI and physical activity) and by physical constraints of the body's systems.

## 10. Implications for obesity

The evidence described above regarding the determination of EI by FFM and RMR and incorporated into a model of appetite control can account for the gradual escalation of body fat via a positive feedback system. During the development of obesity, as FM increases, FFM also increases with an inevitable increment in the drive to eat. At the same time, it can be deduced that the increase in FM will lead to a decrease in inhibition of appetite owing to the onset of leptin and insulin resistance. Consequently, as a person becomes fatter (and also accrues FFM) they will display a stronger drive to eat accompanied by a weakening inhibition, i.e. a reduction in the strength of the signals that suppress eating. Therefore, people living with obesity do not receive any help from their increasing amounts of stored energy (as fat) to constrain their appetite. In fact the opposite is true; appetite self-control becomes more difficult. There is another social implication of the physiological determinants of the drive to eat. It is clearly observed that people with a high BMI (living with obesity) consume more energy than lean people. This is often explained by a lack of self-control, absence of will power or a failure of self-discipline. While the initial causes of overconsumption and weight gain are probably rooted in a range of biological, psychological, social, and environmental pressures, the increases in FFM and FM seen with initial weight gain may exacerbate the progressive escalation of body weight owing to an increase in the tonic drive to eat (increased FFM) and reduced inhibition (increased FM), especially when coupled with a high energy dense diet. This does not mean that a tendency to increase food consumption makes appetite control impossible, but it reveals the nature of the difficulty in exerting a restraining influence on appetite. Recognition of this understanding has implications for the assignment of responsibility in obesity management and for the processes underlying stigma and blame.

It is often written, and said in debate, that the slow increase in body weight over a long period of time is evidence for energy homeostasis or body weight regulation. However, there is nothing in such a gradual process, or the small average increase in weight over the life course, that proves the existence of a deliberate homeostatic control of body weight (or body fat). Such an incremental effect is quite consistent with other mechanisms that produce equilibration, dynamic stability or adaptation. The slow increase in FM and body weight can be conceived as a passive process [78] brought about by the drive to eat, but subject to some limiting processes such as the weak restraining effect of FM and inhibitory influences from the gastrointestinal tract. This account does not require the invoking of a process of regulation [81].

## 11. Conclusion

This review has brought together the findings of approximately 15 years of research from several research groups using a systems approach to throw light on what is known as the field of appetite studies. The focus of this new body of data has been the relationships between body composition, RMR and EI. Separate tonic roles have been proposed for FFM and FM. A strong body of evidence has demonstrated a positive association between FFM and EI, more specifically food consumption and hunger. Direct observations and path analysis have shown that the effect of FFM is mediated by RMR (although direct effects of FFM cannot be ruled out). The most logical interpretation of these findings is that FFM and RMR constitute a tonic source of the drive to eat. This proposal is not consistent with the dogma that food intake serves to regulate body fat, an idea that has dominated appetite research for half a century. Our proposal is that neither body FM nor body weight in general are actively regulated when not challenged by extensive weight or fat loss, but are passively determined as a consequence of the control of food intake. Body composition itself does exert an influence on food intake via multiple direct and indirect effects, but an important feature of the Leeds Model is that both FFM and FM are integrated into the drive and inhibitory processes, respectively, that influence food intake. Given the nature of this Royal Society conference to explore evidence, theory and conjecture, we feel that now is an appropriate time to re-frame the study of appetite separate from notions of fat or weight regulation.

## Data Availability

This article has no additional data.
